# Role played by periaqueductal gray neurons in parasympathetically mediated fear bradycardia in conscious rats

**DOI:** 10.14814/phy2.12831

**Published:** 2016-06-22

**Authors:** Satoshi Koba, Ryo Inoue, Tatsuo Watanabe

**Affiliations:** ^1^ Division of Integrative PhysiologyTottori University Faculty of MedicineYonagoTottoriJapan

**Keywords:** Fear, parasympathetic outflow, periaqueductal gray

## Abstract

Freezing, a characteristic pattern of defensive behavior elicited by fear, is associated with a decrease in the heart rate. Central mechanisms underlying fear bradycardia are poorly understood. The periaqueductal gray (PAG) in the midbrain is known to contribute to autonomic cardiovascular adjustments associated with various emotional behaviors observed during active or passive defense reactions. The purpose of this study was to elucidate the role played by PAG neurons in eliciting fear bradycardia. White noise sound (WNS) exposure at 90 dB induced freezing behavior and elicited bradycardia in conscious rats. The WNS exposure‐elicited bradycardia was mediated parasympathetically because intravenous administration of atropine abolished the bradycardia (*P* < 0.05). Moreover, WNS exposure‐elicited bradycardia was mediated by neuronal activation of the lateral/ventrolateral PAG (l/vlPAG) because bilateral microinjection of muscimol, a GABA_A_ agonist, into the l/vlPAG significantly suppressed the bradycardia. It is noted that muscimol microinjected bilaterally into the dorsolateral PAG had no effect on WNS exposure‐elicited bradycardia. Furthermore, retrograde neuronal tracing experiments combined with immunohistochemistry demonstrated that a number of l/vlPAG neurons that send direct projections to the nucleus ambiguus (NA) in the medulla, a major origin of parasympathetic preganglionic neurons to the heart, were activated by WNS exposure. Based on these findings, we propose that the l/vlPAG‐NA monosynaptic pathway transmits fear‐driven central signals, which elicit bradycardia through parasympathetic outflow.

## Introduction

In mammals, fear is a vital response to threat and potential danger that elicits characteristic patterns of defensive behaviors including freezing. Freezing behavior is a preparatory reflex to escape detection by a predator or potential and/or unknown threats. This reflex behavior in animals and humans was demonstrated to be associated with the heart rate (HR) deceleration or fear bradycardia (Lang and Davis 2006; Hagenaars et al. 2014). In conscious, free‐moving rats, white noise sound (WNS) exposure reportedly induced freezing behavior and caused a remarkable decrease in HR (Yoshimoto et al. 2010). In humans, freezing‐like behavior experimentally induced by pictorial stimuli was shown to be associated with a significant decrease in HR (Hermans et al. 2013). Fear bradycardia is considered a preparatory cardiovascular adjustment for performing the fight or flight action. The decreased HR would extend the response range, from the resting to the maximal HR level, thereby allowing an increase in the response range of cardiac output while conserving energy in the heart muscle (Miki and Yoshimoto 2010; Yoshimoto et al. 2010). Central mechanisms underlying fear bradycardia are poorly understood.

The periaqueductal gray (PAG) in the midbrain is a cell‐dense region that surrounds the midbrain aqueduct and contains longitudinal columns including dorsomedial (dmPAG), dorsolateral (dlPAG), lateral (lPAG), and ventrolateral (vlPAG) subdivisions (Carrive 1993; Bandler and Shipley 1994; Bandler et al. 2000). While the PAG is known to be involved in the expression of specific autonomic cardiovascular changes associated with various emotional behaviors observed during active or passive defense reactions (Carrive 1993; Bandler and Shipley 1994; Bandler et al. 2000; Keay and Bandler 2001; Hagenaars et al. 2014), the precise roles played by the PAG neurons in eliciting fear bradycardia have not been explored. Nevertheless, several lines of previous data led us to hypothesize that activation of lateral and ventrolateral parts of the PAG (l/vlPAG) contributes to the expression of fear bradycardia by increasing parasympathetic activity. In humans, activation of the PAG, as evaluated by blood oxygenation level‐dependent functional MRI, during freezing‐like behavior correlated with bradycardia, suggesting that fear bradycardia accompanies excitation of PAG neurons (Hermans et al. 2013). However, because of the limited spatial resolution of fMRI, the PAG subdivisions that were activated in association with bradycardia during the behavior were not determined. It was also uncertain whether activation of the PAG was a cause of the bradycardia. Another study performing neuronal tracing on rat brains (Ennis et al. 1997) showed that efferent projections originating in the l/vlPAG terminate in close contiguity to cholinergic neurons in the nucleus ambiguus (NA) in the medulla. The NA and area surrounding its compact portion are brain regions that provide parasympathetic innervation of the heart in rats (Stuesse 1982; Chitravanshi and Sapru 2011; Panneton et al. 2014). A functional linkage between the l/vlPAG and parasympathetic nervous system has also been implicated in humans. Direct electrical stimulation of the ventral part of the human PAG reportedly evoked cardiac parasympathetic activation, as assessed by heart rate variability (Pereira et al. 2010). However, it is unclear whether l/vlPAG neurons projecting to the NA are activated by fear, thereby eliciting parasympathetically mediated bradycardia.

The purpose of this study was to elucidate the role played by the PAG neurons in eliciting fear bradycardia through parasympathetic outflow. Firstly, we determined whether fear bradycardia is parasympathetically mediated by examining the effect of intravenous administration of atropine on HR and arterial pressure responses to WNS exposure in free‐moving, conscious rats. We also identified subdivisions in the caudal region of the PAG that play a role in expressing fear bradycardia by investigating the effect of microinjection of muscimol, a GABA_A_ receptor agonist, into the PAG on cardiovascular responses to WNS exposure in rats. In this study, the caudal PAG was the focus of investigation since caudal PAG neurons reportedly play a dominant role in cardiovascular regulation during “passive” defensive behavior (Bandler et al. 2000). Moreover, via the retrograde neuronal tracing combined with immunohistochemistry, we explored the central circuitry that contains PAG neurons and underlies fear bradycardia by mapping the distribution of NA‐projecting, WNS exposure‐excited neuronal cells in the caudal PAG.

## Methods

### Ethical approval

All procedures outlined in this study complied with the Guiding Principles for the Care and Use of Animals in the Fields of Physiological Sciences of the Physiological Society of Japan, and were approved by the Animal Care Committee of Tottori University (reference number: 13‐Y‐47). The experiments were performed on male adult Sprague–Dawley rats (*N* = 37, aged 9–16 weeks, 280–463 g). Rats were housed in standard rodent cages in a temperature‐controlled room (24–25°C) and were regulated on a 12:12 h light–dark schedule. Food and water were made available ad libitum.

### Experiment 1: effect of prior administration of atropine on cardiovascular responses to WNS exposure in conscious, free‐moving rats

Experiment 1 was conducted to determine whether fear bradycardia is parasympathetically mediated. As performed previously (Yoshimoto et al. 2010), rats (*N* = 7) were chronically implanted with arterial and venous catheters for arterial pressure (AP) measurements and drug infusion, respectively. A bipolar electrode for electrocardiography (ECG) was also implanted. Briefly, in rats anesthetized with a mixture of isoflurane (<4%) and oxygen, the arterial catheter was implanted via the tail artery on the ventral side, and the tip of the catheter was placed approximately 10 mm above the aortic bifurcation. The venous catheter was implanted via the right jugular vein, and the tip of the catheter was placed at the junction of the superior vena cava and right atrium. Syringes were connected to the arterial and venous catheters, respectively, and aspiration of blood was taken to indicate correct placement of the catheters. Then, the catheters were filled with a mixture of 0.9% NaCl and heparin (1000 IU/mL). The bipolar electrode was implanted under the skin at midchest level. The catheters and electrode were exteriorized between the ears and protected by a 15‐cm coiled spring. After the surgery, the rats were housed individually. At least 72 h were allowed for recovery before data collection.

Each rat was subjected to two different trials separated by 3 days apart and conducted in random order in which intravenous administration of either normal saline or atropine diluted in saline was followed by WNS exposure. On the day of the experiment, the rats in their home cages were brought to the experimentation room, in which the temperature was maintained at 24–25°C and the ambient noise level was <65 dB. The cage was covered by a blackout curtain with a small window to observe rat behavior. The arterial catheter was attached to a pressure transducer (P23XL; Becton Dickinson & Co., Newark, DE). The bipolar electrode was connected to an AC amplifier (P511; Grass Instruments, Natus Neurology, Inc., Warwick, RI), which was used to amplify the ECG signal. Noise intensity around the cage was measured by placing a sound level metre (TM‐102, Sato Shouji Inc., Kawasaki, Japan). Then, more than 1 h was allowed to pass before the conscious rats were intravenously administered saline (0.2 mL) or atropine solution (100 *μ*g diluted in 0.2 mL saline, Ito et al. 2004). A duration of 5–10 min after administration, the conscious rats were exposed to WNS at 90 dB for 5 min. Throughout the protocol, the rat behavior was monitored visually by the investigator through the small window in the curtain covering the cage. At the end of the entire procedure, the rats were deeply anesthetized with 5% isoflurane in oxygen and killed via an intravenous infusion of saturated potassium chloride solution (1 mL).

### Experiment 2: effect of muscimol microinjected bilaterally into the PAG on cardiovascular responses to WNS exposure in conscious, free‐moving rats

In rats used for Experiment 2 (*N* = 17), the guide cannulae for microinjection into the caudal PAG were chronically implanted. The rats were anesthetized with a mixture of 2–4% isoflurane and oxygen, intubated, and artificially ventilated (SN‐480‐7, Shinano Co., Tokyo, Japan). Then, they were placed in a stereotaxic apparatus (900LS; David Kopf Instruments, Inc., Tujunga, CA) with the incisor bar positioned at 3.5 mm below the interaural line. In accordance with a previous study (de Menezes et al. 2008), target coordinates for the caudal PAG were set at 7.0–8.0 mm posterior, 0.7 mm lateral, and 4.8–5.3 mm ventral relative to the bregma. The guide cannulae (Plastics One, Anaheim, CA) were secured by two screws and dental acrylic. Dummy cannulae were inserted to the guides. The rats were housed individually for recovery.

Following 11–30 days of recovery of the rats, they were processed to the next surgery to implant arterial and venous catheters and an electrode for recording ECG, as conducted in Experiment 1. At least 72 h were allowed for recovery before data collection.

In the protocols for Experiment 2, bilateral microinjection of either normal saline or muscimol solution into the caudal PAG was followed by WNS exposure. Each rat was subjected to two different trials separated by 3 days and conducted in random order. As performed in Experiment 1, the rats were prepared in the experimentation room for data collection. Additionally, a bilateral internal injector (33 gauge, 1.0 mm longer than the guide cannula; Plastics One, Anaheim, CA) was placed into the guide cannulae and connected to 2 *μ*L Hamilton syringes with Teflon tubing. The syringes were mounted on manual microinjectors (IM‐3; Narishige, Tokyo, Japan). More than 1 h was allowed after the placement of the bilateral internal injector. Following 30–40 min after bilateral microinjection of saline (100 nL) or muscimol solution (300 pmol diluted in 100 nL saline) into the PAG, the conscious rats were exposed to WNS at 90 dB for 5 min. The dosage of muscimol solution was chosen in accordance with a previous study (de Menezes et al. 2008), reporting that prior microinjection of muscimol into the dlPAG of conscious rats significantly reduced pressure and tachycardia responses to air‐jet stress. After all the observations had been conducted, the rats were deeply anesthetized with 5% isoflurane in oxygen, and microinjection sites were marked using India ink for histological verification. Then, the animals were killed via an intravenous infusion of 1 mL saturated potassium chloride solution, and their brains were removed and coronally cryostat‐sectioned to verify the sites in which India ink was microinjected. Injection sites were approximated using the atlas of Paxinos and Watson (2007).

### Experiment 3: retrograde tract tracing and immunohistochemistry to map the distribution of NA‐projecting, WNS exposure‐activated neurons in the caudal PAG

Our pilot study (Inoue et al. 2015) demonstrated that WNS exposure to conscious, free‐moving rats increased expression of Fos protein, a marker of neural activation (Sagar et al. 1988), in central cardiovascular regions, including the PAG and NA. Experiment 3 was performed to examine the distribution of central monosynaptic pathways from the PAG to NA, which are activated by WNS exposure.

To label neuronal cells sending projections to the NA of rats, retrograde tracing was performed in rat brains with a retrograde tracer cholera toxin subunit B (CTb). Rats (*N* = 13) were anesthetized with a mixture of oxygen and isoflurane (<4%), intubated, artificially ventilated, and placed in the stereotaxic apparatus. The neck muscles were dissected and the dura matter was incised to expose the dorsal surface of the medulla. The site of the rat NA was determined as previously described (Chitravanshi and Sapru 2011). Briefly, a glass micropipette filled with l‐glutamate solution (10 mmol/L in saline) was inserted into the brainstem at an angle vertical to the dorsal surface with the aid of the microscope. Initial coordinates for the NA were 0.3 mm rostral to 0.5 mm caudal, and 1.8–2.0 mm lateral to the calamus scriptorius and 2.0–2.4 mm ventral to the dorsal medullary surface. The location of the NA was functionally identified by observing a decrease in HR when glutamate (46.0 nL) was pressure ejected using a calibrated microinjection system (Nanoject II; Drummond Scientific, Co., Broomall, PA). Then, the rats received a pressure microinjection of Alexa‐594‐conjugated CTb (1.0 mg in 1 mL phosphate‐buffered saline [PBS, pH 7.4], 32.2 nL ×3, 2 min were allowed between injections) (Molecular Probes, Eugene, OR) into the NA unilaterally. After the surgery, the rats were housed individually. A duration of 9–11 days were allowed before the protocol for Experiment 3.

On the protocol day, a subset of rats that had received CTb (*N* = 7) were exposed to WNS for 30 min at 90 dB in a similar manner as described in Experiment 1–2. As a control, another subset of rats (*N* = 6) were brought to the experimentation room but not exposed to WNS. Two hours after the end of WNS exposure or the control period, the rats were deeply anesthetized with 5% isoflurane in oxygen and perfused transcardially with saline followed by 4% paraformaldehyde in 0.1 mol/L PBS (pH 7.4). Brains were removed and postfixed for 4–12 h in 4% paraformaldehyde and then transferred to a 30% sucrose solution at 4°C for 24–48 h. Then, the brains were embedded in optimal cutting temperature compounds (Sakura Finetek, Tokyo, Japan) and coronally cryostat‐sectioned at 35 *μ*m (Leica CM1900; Wetzlar, Germany). Brain sections containing the PAG were prepared at 6.6, 7.2, 7.8, and 8.4 mm caudal to the bregma according to the atlas of Paxinos and Watson (2007). The sections containing the NA at 13.5–14.0 mm caudal to the bregma were also prepared.

The brain sections were subjected to CTb and Fos immunofluorescence staining to enhance CTb‐positive signals and assess activated neuronal cells, respectively, in accordance with a previously described method (Madden 2012). Briefly, the tissue sections were washed in PBS, and incubated in the antibody dilution solution (PBS containing 0.3% Triton X‐100, 2.5 g/L lambda carrageenan, 200 mg/L NaN3, 10 mL/L normal donkey serum) for 2 h at room temperature. Then, the sections were incubated in the primary antibody solution: goat anti‐CTb (0.1 *μ*L/mL in antibody dilution solution, List Biological Laboratories, Campbell, CA) and rabbit anti‐c‐Fos (2 *μ*L/mL in antibody dilution solution, Cell Signaling Technology, Danvers, MA) overnight on a shaker table at 4°C. The sections were rinsed in PBS containing 0.03% Triton X‐100 (2 × 10 min), incubated in the secondary antibody solution: donkey anti‐rabbit Alexa Fluor 488 (5 *μ*L/mL in antibody dilution solution, Molecular Probes) and donkey anti‐goat Alexa Fluor 594 (5 *μ*L/mL in antibody dilution solution, Molecular Probes) in the dark for 1 h at room temperature, and rinsed in PBS (2 × 10 min). Then, they were mounted on slides and coverslipped using Prolong Gold antifade reagent (Molecular Probes). The sections were observed under a fluorescence microscope (BZ‐9000, Keyence, Osaka, Japan). Brain regions were approximated using the atlas of Paxinos and Watson (2007).

### Data and statistical analyses

During data collection in Experiment 1 or 2, arterial pressure, ECG, and HR data were continuously displayed on a computer monitor and stored on the hard disk at a sampling rate of 1 kHz through an analogue‐digital interface (PowerLab/8s, AD Instruments, Dunedin, New Zealand). HR was calculated beat to beat with detection of the time between successive R waves in the ECG. Data were expressed as mean ± SEM. Statistical significances were assessed using a paired *t*‐test, two‐sample *t*‐test or repeated measures ANOVA with post hoc testing as appropriate. The statistical test employed for figure presentation is described in the Figure Legends. The level of significance was set at *P* < 0.05.

## Results

### Characteristics of behavior and cardiovascular changes during 5 min of WNS exposure

**Figure 1:**
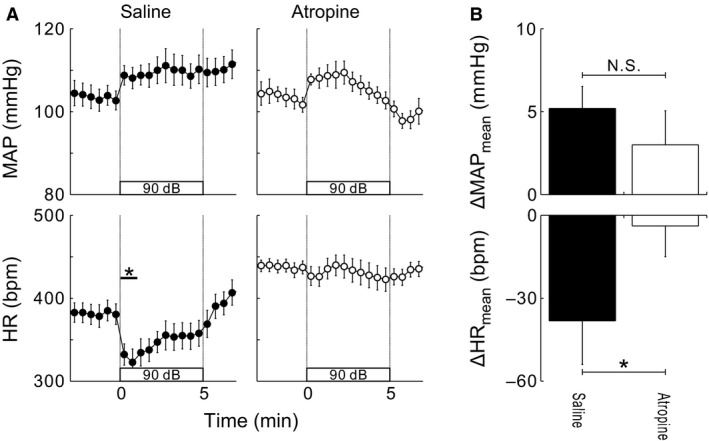
(A) Thirty second averaged time courses of mean arterial pressure (MAP) and heart rate (HR) while conscious, free‐moving rats (*N* = 7) were exposed to 5 min of white noise sound (WNS) at 90 dB. At 5–10 min prior to the onset of WNS exposure, saline (0.2 mL, left) or atropine (100 *μ*g diluted in 0.2 mL of saline, right) was intravenously infused. Values are mean ± SEM. *P < 0.05 versus baseline, detected by Dunnett's post hoc test following one‐way repeated‐measures ANOVA. (B) Comparisons of 5‐min averaged changes from baseline in MAP and HR between saline‐ and atropine‐infused trials. N.S.: no significance. *P < 0.05 saline‐infused versus atropine‐infused, as detected by a paired *t*‐test.

**Figure 2:**
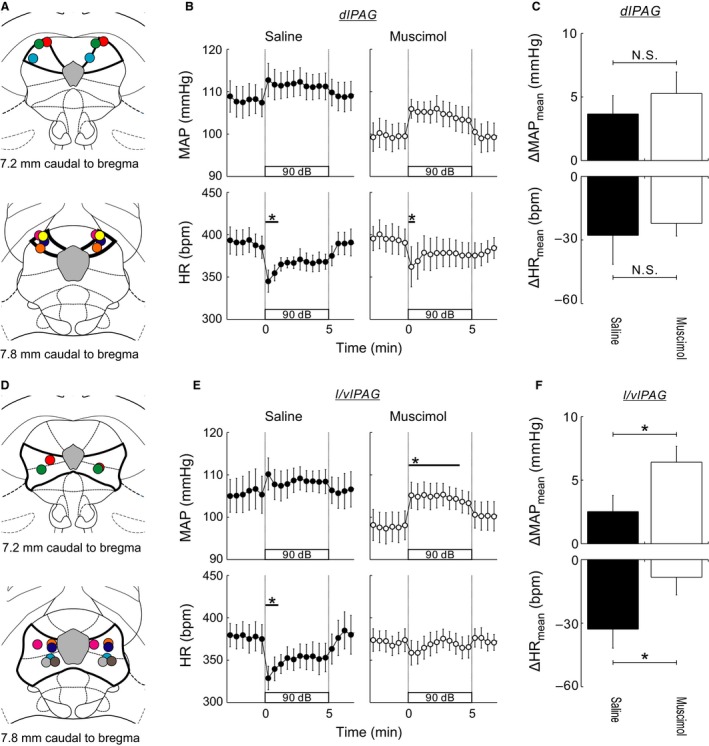
(A and D) Microinjection sites mapped on standard sections from Paxinos and Watson (2007). These sites were marked with injected Indian ink. In seven of the 15 rats (A), the injections were considered to be made in the dorsolateral periaqueductal gray (dlPAG). In the other eight rats (D), the injectates were administered in the lateral/ventrolateral PAG (l/vlPAG). (B and E) Thirty second averaged time courses of MAP and HR while conscious, free‐moving rats were exposed to 5 min of WNS at 90 dB. At 30–40 min prior to the onset of WNS exposure, saline (100 nL) or muscimol (300 pmol in 100 nL of saline) was bilaterally microinjected into the dlPAG (B, *N* = 7) or l/vlPAG (E, *N* = 8). Values are mean ± SEM. *P < 0.05, versus baseline, detected by Dunnett's post hoc test following one‐way repeated‐measures ANOVA. (C and F) Comparisons of 5 min averaged changes from baseline in MAP and HR between saline and muscimol microinjections into the dlPAG (C) or l/vlPAG (F) prior to WNS exposure. N.S.: no significance. *P < 0.05, saline‐microinjected versus muscimol‐microinjected, as detected by a paired *t*‐test.

Conscious, free‐moving rats displayed freezing behavior throughout the 5‐min period of WNS exposure at 90 dB. Freezing was defined as the absence of any movement, excluding breathing and whisker twitching, as performed in previous studies (Bruchey et al. 2010). Data obtained from rats that displayed behaviors other than freezing (e.g., “flight” behavior and/or walking) during the WNS exposure period were not included in the analyses. In rats that did not receive pharmacological treatments (control trials), WNS exposure did not have remarkable effects on AP, but it markedly decreased HR (Figs. 1 and 2), as reported previously (Yoshimoto et al. 2010).

### Experiment 1: effect of prior administration of atropine on cardiovascular responses to WNS exposure in conscious, free‐moving rats

In seven rats, the effect of prior administration of atropine on cardiovascular responses to WNS exposure was examined. Atropine administration had no effect on baseline mean AP (MAP), but it significantly elevated baseline HR (Table 1). WNS exposure 5–10 min after intravenous saline administration did not significantly change AP, but it elicited bradycardia. WNS exposure 5–10 min after atropine was administered did not change either AP or HR from baseline (Fig. 1).

**Table 1:** Baseline mean arterial pressure (MAP) and heart rate (HR) in Experiment 1 and 2

	Experiment 1		Experiment 2			
	Saline, IV	Atropine, IV	Saline, dlPAG	Muscimol, dlPAG	Saline, l/vlPAG	Muscimol, l/vlPAG
Baseline MAP, mmHg	104 ± 3	104 ± 2	108 ± 4	99 ± 3	106 ± 4	98 ± 4
Baseline HR, beats per min	383 ± 13	435 ± 8^a^	392 ± 15	397 ± 18	381 ± 17	375 ± 13

Baseline data were obtained by 5‐min averaged values immediately prior to WNS exposure.

a *P* < 0.05 versus control saline protocol in each rat group, as detected by a paired *t*‐test. Values are mean ± SEM.

### Experiment 2: effect of muscimol microinjection into the PAG on cardiovascular responses to WNS exposure in conscious, free‐moving rats

In 17 rats in which the bilateral guide cannulae had been manipulated within the brain, the effect of pharmacological inhibition of neuronal activation in the caudal PAG on WNS exposure‐elicited cardiovascular changes was examined (Fig. 2). In two of the 17 rats, data collection was discontinued because they displayed flight behavior/walking when exposed to WNS. The remaining 15 rats displayed freezing behavior throughout the 5‐min WNS exposure in both saline and muscimol trials. In seven of the 15 rats, the tips of cannulae for microinjection were located within the dlPAG at 7.0–8.0 mm caudal to the bregma, as dyed post hoc by microinjected India ink (Fig. 2A). Muscimol microinjection into the dlPAG had no significant effect on baseline MAP or HR (paired *t*‐test, Table 1). WNS exposure 30–40 min after bilateral microinjection of either saline or muscimol into the dlPAG did not significantly change AP from baseline, but it elicited bradycardia (Fig. 2B). Bradycardia in response to WNS exposure was not significantly different between the saline and muscimol trials (Fig. 2C).

In eight of the 15 rats, the tips of cannulae for microinjection were located within the l/vlPAG at 7.0–8.0 mm caudal to the bregma (Fig. 2D). Muscimol microinjection into the l/vlPAG did not have significant effects on baseline MAP or HR (Table 1). At 30–40 min after bilateral microinjection of saline into the l/vlPAG, WNS exposure did not change AP from baseline, but it significantly decreased HR (Fig. 2E). By contrast, WNS exposure after muscimol microinjection into the l/vlPAG significantly elevated AP, whereas HR was not changed from baseline. The MAP and HR responses to WNS exposure were significantly different between the saline and muscimol trials (Fig. 2F).

### Experiment 3: retrograde tract tracing and immunohistochemistry to map the distribution of NA‐projecting, WNS exposure‐activated neurons in the caudal PAG

**Figure 3:**
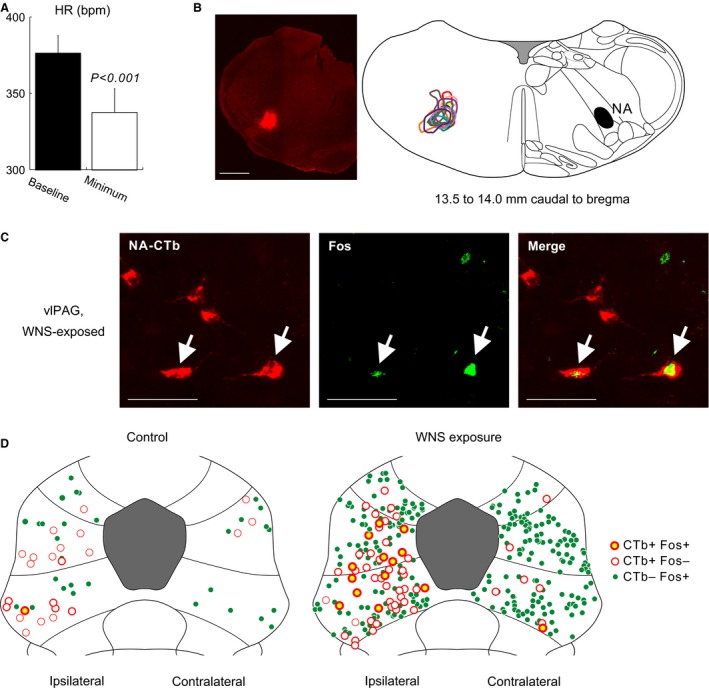
(A) HR at baseline and minimum HR following microinjection of l‐glutamate (10 mmol/L in saline, 46.0 nL) into the nucleus ambiguus (NA) in isoflurane‐anesthetized rats (*N* = 13). Values are mean ± SEM. A significant difference (*P* < 0.001) was detected by a paired *t*‐test. (B) Left: a representative photomicrograph of the cholera toxin subunit B (CTb) injection site. Scale bar in the micrograph: 1 mm. Right: maximal spreads of the injected CTb in 13 cases outlined on a schematic section adapted from Paxinos and Watson (2007) (Right). The black filled area in the illustration indicates the contralateral NA. (C) Representative photomicrographs showing Fos immunoreactivity in CTb‐labeled cells (arrows) following 30 min of WNS exposure at 90 dB. The cells shown in the photomicrographs were located in the vlPAG (7.8 mm caudal to bregma) ipsilateral to the NA, into which CTb was microinjected. Scale bars: 50 *μ*m. (D) As an example, the distribution of NA‐projecting, CTb‐labeled cells with (yellow‐filled, red‐outlined circle) and without (white‐filled, red‐outlined circle) Fos immunoreactivity as well as Fos immunoreactive cells without CTb positive signals (green‐filled circle) in control (Left) and WNS‐exposed (Right) rats is plotted on schematic sections of the PAG at 7.8 mm caudal to bregma (Paxinos and Watson 2007).

In 13 rats anesthetized with isoflurane, the site of the unilateral NA was determined by confirming a significant bradycardia response to microinjection of glutamate (−39 beats per min from baseline, on average) (Fig. 3A). Then, a retrograde tracer CTb was microinjected into the NA. The injected location was found post hoc in the coronal section at 13.5–14.0 mm caudal to the bregma (Fig. 3B). A duration of 9–11 days later, seven of the 13 rats were subjected to 30 min of WNS exposure at 90 dB, displaying freezing behavior. During the WNS exposure, “active” defensive behaviors such as flight behavior or walking were not found more than 1 min in total. The remaining six rats served as controls that were not exposed to WNS.

**Figure 4:**
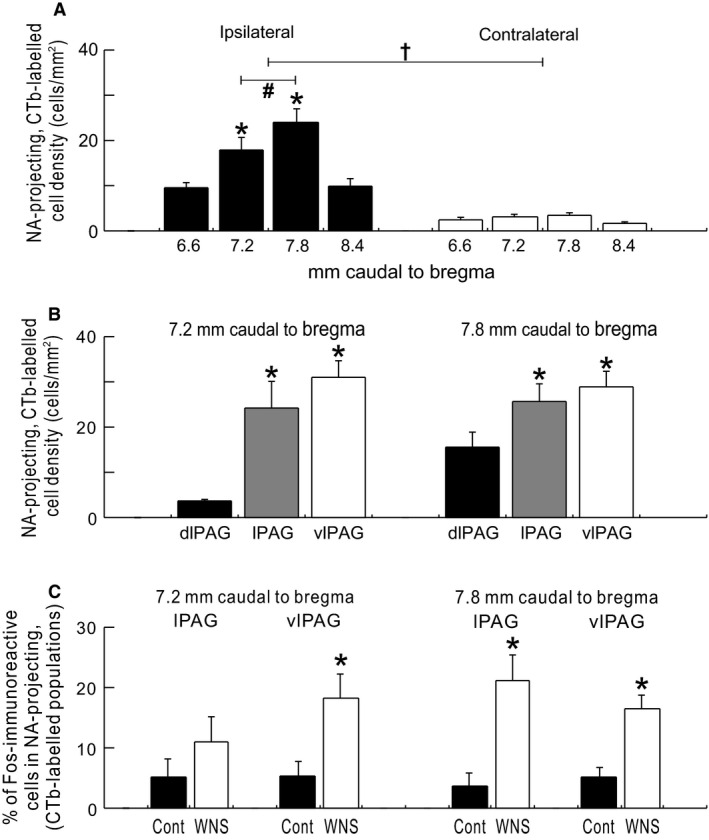
(A) Comparisons of NA‐projecting, CTb‐labeled cells density in the PAG subdivisions (dlPAG, lPAG, and vlPAG) between the ipsilateral and contralateral sides of the NA, into which CTb was microinjected, and between the bregma levels on each side (*N* = 13). Values are mean ± SEM. *P < 0.05, versus 6.6 and 8.4 mm caudal to bregma. ^#^ *P* < 0.05, 7.2 versus 7.8 mm caudal to bregma. ^†^ *P* < 0.05, ipsilateral versus contralateral. Significant differences were detected by Tukey's post hoc test following two‐way repeated‐measures ANOVA (bregma level × brain side). (B) Comparisons of NA‐projecting, CTb‐labeled cell density among dlPAG, lPAG, and vlPAG (ipsilateral side) at 7.2 and 7.8 mm caudal to bregma (*N* = 13). *P < 0.05, versus dlPAG, detected by Tukey's post hoc test following one‐way repeated‐measures ANOVA. (C) Comparisons between control (*N* = 6) and WNS‐exposed (*N* = 7) rats of the percentages of Fos‐immunoreactive cells in NA‐projecting, CTb‐labeled populations in the lPAG and vlPAG (ipsilateral side) at 7.2 and 7.8 mm caudal to bregma. *P < 0.05, versus control, as detected by an unpaired *t*‐test.

In the 13 rats, a significant number of NA‐projecting, CTb‐labeled neurons were found in the PAG at 6.6, 7.2, 7.8, and 8.4 mm caudal to the bregma (Fig. 3C and D), and dominantly distributed in the ipsilateral PAG to the NA into which CTb had been microinjected, compared with those at the contralateral side at each bregma level (Fig. 4A). In the ipsilateral PAG, moreover, a higher density of NA‐projecting, CTb‐labeled neurons was found at bregma −7.2 and −7.8 mm than that at bregma −6.6 or −8.4 mm (Fig. 4A). In the ipsilateral PAG at bregma −7.2 and −7.8 mm, NA‐projecting, CTb‐labeled neurons were dominantly expressed in the lPAG and vlPAG compared with those in the dlPAG (Fig. 4B).

Expression of Fos was also examined in six controls and seven WNS‐exposed rats. As we previously observed (Inoue et al. 2015), WNS exposure in conscious rats resulted in a remarkable increase in Fos expression in the caudal PAG (Fig. 3D and Table 2), suggesting that WNS exposure is a cause of neuronal excitation in the PAG. In the caudal PAG of WNS‐exposed rats, a number of NA‐projecting, CTb‐labeled neuronal cells with Fos immunoreactivity were found (Fig. 3C and D). In the vlPAG at bregma −7.2 mm and the lPAG and vlPAG at bregma −7.8 mm of WNS‐exposed rats, the percentages of Fos‐immunoreactive cells in the NA‐projecting, CTb‐labeled populations (17–21% on average) were significantly higher than those of control rats (4–5% on average) (Fig. 4C). In addition, in the ipsilateral l/vlPAG of WNS‐exposed rats, the percentages of NA‐projecting, CTb‐labeled cells in the Fos‐immunoreactive populations were 5 ± 2% (lPAG) and 12 ± 2% (vlPAG) at bregma −7.2 mm, and 10 ± 2% (lPAG) and 14 ± 3% (vlPAG) at bregma −7.8 mm.

**Table 2:** Comparison between control (*N* = 6) and WNS‐exposed (*N* = 7) rats of Fos expression in the subdivisions of the PAG

mm caudal from bregma	PAG subdivisions	Fos density, cells per mm^2^	
		Control	WNS exposed
6.6	dlPAG	35.1 ± 5.6	94.6 ± 16.9^a^
	lPAG	25.3 ± 4.4	74.3 ± 15.6^a^
	vlPAG	16.9 ± 3.8	55.9 ± 12.5^a^
7.2	dlPAG	29.4 ± 4.5	81.6 ± 1.5^a^
	lPAG	29.2 ± 3.3	73.6 ± 14.3^a^
	vlPAG	32.6 ± 5.8	60.0 ± 13.1^a^
7.8	dlPAG	23.7 ± 9.5	78.4 ± 16.6^a^
	lPAG	34.6 ± 7.9	61.3 ± 12.3^a^
	vlPAG	24.0 ± 6.4	52.5 ± 8.9^a^
8.4	lPAG	19.5 ± 5.1	55.0 ± 13.2^a^
	vlPAG	11.9 ± 3.0	54.9 ± 10.0^a^

a *P* < 0.05 versus control at each PAG subdivision, as detected by an unpaired *t*‐test. Values are mean ± SEM. The dlPAG is not present at 8.4 mm caudal to the bregma, according to the atlas of Paxinos and Watson (2007).

## Discussion

**Figure 5:**
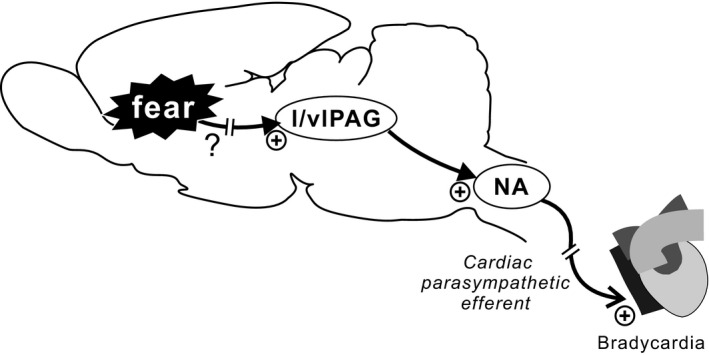
This study proposes a central circuit responsible for the expression of fear bradycardia. Fear signals excite l/vlPAG neurons which provide a direct input to the NA. Then, parasympathetically mediated bradycardia is elicited. The upstream circuits that send projections to the l/vlPAG, thereby underlying fear bradycardia are currently unknown.

White noise sound exposure in conscious, free‐moving rats induced freezing behavior, an index of fear, and elicited bradycardia, as previously reported (Yoshimoto et al. 2010). The main findings of this study are as follows. Firstly, prior systemic administration of atropine abolished bradycardia in response to WNS exposure. This result demonstrates that WNS exposure‐elicited bradycardia is parasympathetically mediated. Secondary, prior microinjection of muscimol into the caudal l/vlPAG suppressed bradycardia in response to WNS exposure. Of note, muscimol preliminarily microinjected into the dlPAG did not change the bradycardia. These results demonstrate that neuronal excitation in the l/vlPAG, but not in the dlPAG, during WNS exposure in rats is a cause of the bradycardia. Moreover, retrograde neuronal tracing experiments combined with immunohistochemistry illustrated: (1) that a majority of neuronal cells labeled by CTb, which had been unilaterally microinjected into the NA, was found on the ipsilateral side of the caudal (7.0–8.0 mm caudal to the bregma) PAG, (2) that on the ipsilateral side of the PAG, NA‐projecting, CTb‐labeled neurons were densely distributed in the l/vlPAG compared with the dlPAG, and 3) that in the ipsilateral l/vlPAG of the WNS‐exposed rats, 15–20% of the NA‐projecting, CTb‐labeled neurons were also immunoreactive to Fos. These results demonstrate that a number of caudal l/vlPAG neurons that send direct projections to the ipsilateral NA were activated by WNS exposure. Taken together, these results suggest that the l/vlPAG‐NA monosynaptic pathway transmits fear‐driven central signals, which elicit bradycardia through parasympathetic outflow (Fig. 5).

Several studies have demonstrated that autonomic responses associated with freezing behavior include simultaneous activation of sympathetic and parasympathetic components (Nijsen et al. 1998; Carrive 2006; Hermans et al. 2013). Yoshimoto et al. (2010) provided evidence for sympathoexcitation in response to WNS exposure in rats by directly measuring renal and lumbar sympathetic nerve activities. Based on their observation and the present finding of the effect of atropine administration to abolish the bradycardia response, WNS exposure is considered to evoke both sympathoexcitation and parasympathoexcitation. Sympathoexcitation directed to the heart might also be evoked by WNS exposure. Although the effect of cardiac sympathetic nerve activity on HR was not assessed in this study, cardiac sympathoexcitation might play a role in inhibiting WNS exposure‐elicited bradycardia.

Besides WNS exposure, freezing behavior can also be induced in rats by other emotional stresses such as reexposure to a footshock chamber (Walker and Carrive 2003; Carrive 2006) and social defeat stress (Kataoka et al. 2014). However, these stresses reportedly caused tachycardia instead of bradycardia. This discrepancy may be explained by various patterns of balances between sympathetic and parasympathetic activation during freezing behavior across the studies (Hagenaars et al. 2014). Moreover, differences in paradigms for inducing freezing behavior between the studies might have resulted in minor or major variances in expression of the behavior (Hagenaars et al. 2014). Because this study aimed to investigate central mechanisms underlying fear bradycardia, we employed WNS exposure, which is probably interpreted in rats as an unknown threat and which reportedly induces freezing behavior accompanied with a notable bradycardia (Yoshimoto et al. 2010).

As stated, evidence indicates that the PAG plays a pivotal role in autonomic cardiovascular changes associated with various emotional behaviors during passive (e.g., freezing or immobility) or active (e.g., fight or flight) defense reactions (Carrive 1993; Bandler and Shipley 1994; Bandler et al. 2000; Keay and Bandler 2001; Dampney et al. 2013; Hagenaars et al. 2014). Further, the ventrolateral part of the PAG has been demonstrated to contribute to the expression of passive defensive behavior‐associated cardiovascular changes such as decreased heart rate (Bandler et al. 2000). This study strengthens this notion by demonstrating the suppressive effect of muscimol microinjection into the l/vlPAG, but not dlPAG, on WNS exposure‐elicited bradycardia. Nevertheless, mechanisms by which the ventrolateral part of the PAG contributes to the expression of autonomic responses during passive defensive behavior were previously explained by the ability of the l/vlPAG to reduce sympathetic activity by inhibiting “sympathoexcitatory” activity of rostral ventrolateral medulla or by exciting “sympathoinhibitory” activity of caudal ventrolateral medulla (Keay and Bandler 2001). By contrast, little attention has been paid to the function of the l/vlPAG to modulate parasympathetic outflow during emotional behaviors. This study uncovers a novel role played by the l/vlPAG in increasing parasympathetic outflow directed to the heart, thereby contributing to the expression of fear bradycardia.

The ventrolateral part of the PAG has previously been suggested to play an integrative role in generating distinct patterns of not only autonomic but also motor responses associated with freezing behavior (LeDoux et al. 1988; Carrive et al. 1997; Walker and Carrive 2003). Recent evidence identified a direct pathway linking the vlPAG to the cerebellar cortex, activation of which increases alpha motor activity, thereby contributing to the induction of freezing behavior in rats (Koutsikou et al. 2014). As stated in the Results, 10–15% of Fos‐immunoreactive vlPAG cells of WNS‐exposed rats were colabeled with CTb, suggesting otherwise that a number of the vlPAG neuronal cells were not involved in parasympathetic regulation through the NA. A portion of such neuronal cells might play a role in motor control to induce freezing behavior. Thus, prior microinjection of muscimol into the l/vlPAG might alter characteristics of motor responses during WNS exposure such as recruitment pattern of motor units, although freezing behavior was induced throughout the WNS exposure. Such altered motor responses might indirectly contribute to the suppression of the bradycardia response after pharmacological denervation of the l/vlPAG. Further detailed investigation is needed to test this possibility.

Previously, the lPAG was stated to be functionally involved in the expression of “active” defensive behavior‐associated cardiovascular changes, including elevated blood pressure and HR, through sympathetic outflow (Bandler and Shipley 1994; Bandler et al. 2000; Keay and Bandler 2001; Dampney et al. 2013). Conversely, the previous (Ennis et al. 1997) and present studies indicated that the rat lPAG neurons send direct efferent projections to the NA, suggesting that activation of the lPAG modulates parasympathetic outflow directed to the heart. Moreover, this study demonstrates that the lPAG plays a role in eliciting parasympathetically mediated fear bradycardia, as does the vlPAG, by revealing the effect of muscimol microinjection and the distribution of cells double‐labeled by CTb and Fos in the l/vlPAG. Collectively, the lPAG is considered to play multiple roles in generating various patterns of autonomic responses seen during either active or passive defensive behaviors. It is further postulated that the lPAG may contain both “sympathoexcitatory” neurons and “parasympathoexcitory” neurons and/or neurons that may trigger increases in both sympathetic and parasympathetic outflows. The functional and anatomical characteristics of lPAG neuronal cells are of interest, and they should be further investigated.

By preliminarily microinjecting muscimol into the l/vlPAG, MAP elevated significantly in response to WNS exposure (Fig. 2E). This muscimol effect is likely due to its action to suppress the WNS exposure‐elicited bradycardia, thereby helping cardiac output increase. Nonetheless, the difference in WNS exposure‐elicited MAP changes between the saline and muscimol trials was <5 mmHg in average, if any (Fig. 2F).

In this study, the effect of muscimol microinjection into the dlPAG on bradycardia in response to WNS exposure was slight. Moreover, the distribution of NA‐projecting, CTb‐labeled cells in the dlPAG was low. These results suggest that activation of the dlPAG does not play a role in parasympathetically mediated fear bradycardia. The dlPAG has been considered crucial for the expression of active behavioral responses through sympathetic outflow (Bandler et al. 2000; Keay and Bandler 2001; Green et al. 2005; Dampney et al. 2013). Activation of the dlPAG has also been suggested to play an important role in mediating exercise‐elicited cardiorespiratory responses (Green et al. 2005; Paterson 2014). Because of the sympathoexcitatory effects induced by the dlPAG and the present finding of Fos‐immunoreactive cell upregulation due to WNS exposure (Table 2), WNS exposure‐evoked sympathoexcitation in rats (Yoshimoto et al. 2010) might be hypothetically a result of, at least in part, excitation of the dlPAG neurons. However, the present experiment revealed that muscimol microinjection into the dlPAG had no effect on either HR or MAP in response to WNS exposure. Thus, the roles the dlPAG neurons play in the expression of fear‐driven autonomic responses to an unknown threat such as WNS exposure, including both sympathetic and parasympathetic components, are likely minor.

Brain areas upstream of the l/vlPAG underlying fear bradycardia have yet to be studied (Fig. 5). Nevertheless, several areas are potentially hypothesized based on previous findings. The amygdala, known as an essential region for both innate and learned fear (Calder et al. 2001), plays an important role in inducing freezing behavior as a fear response (LeDoux et al. 1988; Blair et al. 2005). It was also found that central amygdala nucleus projections preferentially terminate in the l/vlPAG (Rizvi et al. 1991). These may hypothesize that direct connections from central amygdala nucleus to the l/vlPAG play a role in triggering fear bradycardia, although this hypothesis remains to be investigated. Moreover, other descending limbic systems to the l/vlPAG reportedly include rostral prelimbic cortex (Floyd et al. 2000), agranular insular cortex (Floyd et al. 2000), and anterior (Semenenko and Lumb 1999) and lateral (Behbehani et al. 1988) hypothalamus. Nevertheless, it is unknown whether these areas are involved in the expression of fear bradycardia via the l/vlPAG.

Study limitations need to be kept in mind when interpreting the data collected in the retrograde neuronal tracing experiments. Although the sites for CTb injection were targeted to the NA and determined by observing the bradycardia in response to glutamate administration, it is unclear if CTb injected was adequately administered to the area in which cell bodies of parasympathetic preganglionic cardiac motoneurons were located. The rat NA is shown to not only provide parasympathetic innervation of the heart (Stuesse 1982; Chitravanshi and Sapru 2011) but also include esophageal, pharyngeal, and laryngeal motoneurons (Kobler et al. 1994). Moreover, recent evidence demonstrates that rat cardiac motoneuron cell bodies are dominantly located in the external formation of the NA, especially its ventral side (Panneton et al. 2014). Therefore, this study design cannot exclude the possibility that a number of CTb‐labeled l/vlPAG neurons send projections to other neuronal cells than cardiac motoneurons. It is also uncertain that how much CTb‐labeled l/vlPAG neurons are directly projected to the parasympathetic preganglionic cardiac motoneurons. A recently established neurotracing strategy, termed TRIO (tracing relationship between input and output) method, enables transsynaptic input tracing from specific subsets of neurons based on their projections (Schwarz et al. 2015). Future studies using the TRIO method may determine PAG neuronal populations which send direct efferent projections to cardiac parasympathetic preganglionic neurons.

Although we propose that the l/vlPAG‐NA monosynaptic pathway is a component of the central circuitries underlying fear bradycardia (Fig. 5), functional roles played by this pathway remain to be clarified. In this regard, current optogenetics technology achieves selective excitation/inhibition of a defined neural pathway (Yizhar et al. 2011; Kataoka et al. 2014). Experiments to examine the effect of optogenetic control of l/vlPAG‐NA pathway activity on fear bradycardia may address this issue. Such studies are needed to better understand the central circuitries underlying fear autonomic responses.

In conclusion, the results demonstrate that: (1) WNS exposure in conscious rats caused bradycardia through parasympathetic outflow, (2) WNS exposure‐elicited bradycardia was mediated by activation of the l/vlPAG, but not dlPAG, and (3) a number of l/vlPAG neurons that send monosynaptic projections to the NA were activated by WNS exposure. Taken together, we suggest that fear‐driven central signals are transmitted to the l/vlPAG‐NA pathway, thereby eliciting bradycardia through parasympathetic outflow. It is noted that fear recognition and central neural response to fear stimuli are enhanced in patients with emotional disorders such as depression (Sheline et al. 2001; Bhagwagar et al. 2004; Monk et al. 2008). In most cases, patients with major depression exhibit autonomic cardiovascular imbalances, which may indicate a predisposition to cardiovascular disease (Kamphuis et al. 2007; Koschke et al. 2009). Central mechanisms uncovered in this study may play a role in the development of autonomic dysfunction under psychopathological conditions. Although the present experiments were conducted in rats and therefore cannot readily be generalized to humans, this report may open perspectives for translational research into the central circuitries linking fear coping with autonomic cardiovascular homeostasis – potentially underlying the etiology of autonomic dysfunction in emotional disorders.

## Conflict of Interest

No conflicts of interests are declared by the authors.
